# Harvey

**Published:** 1864-10

**Authors:** 


### *Harvey.*

In the year 1559, a Doctor of Medicine of the University of Oxford, named John Geynes, was cited before the Royal College of Physicians of London, for impugning the infallibility of Galen. He was accused of having publicly stated that Galen had erred, whereupon he was required to bring forward the points in which he had discovered the errors, only one month being allowed him to prepare the list. "Præceptum est Joanni Genese gratioso alioqui et non impudento viro, ut scriptu collegio ex hibeat omnia ea Galeni loca (intra mensem unum) quibus cum enasse, et vulgo et apud doctos, ac etiam coram universe collegio in solemnibus comitiis congregato dicere, hominem non predebat." Such is the literal record of the fact in the annals of the college.

Dr. Geynes was unable to make out a good case against Galen; and having, by the direction of the president, been summoned through the Sheriff's Court of London either to fulfil the behe-ts of the college or to be conveyed to prison, he clearly understood that he and not Galen had been at fault. Whereupon "he most honestly confessed that he repented of his heresies; that he had proposed futile assertions; that he had not been sufficiently circumspect; that he had not compared the passages of Galen carefully enough; that he had not inquired into his meaning; that he had not understood the sense; that he had not quoted his words faithfully; that he had not shown reverence to Galen, and that he had falsely accused him." Such full confession merited plenary indulgence. The college pardoned the culprit, and ratified the fact by admitting Dr. Geynes to the fellowship in the very next year after the commission of the flagrant delinquency which has just been detailed.

The name of Dr. Geynes is otherwise in no way distinguished from a long list of unremembered Fellows of that body. There is no record of the accusations which Dr. Geynes brought against Galen; possibly we might regard them as futile as they appeared to the college in 1559. We do not know whether he in any way anticipated the great discoveries which in less than a century were endorsed by the same college, and which undermined so much of what, upon the authority of Galen, had been regarded as irrefragible. But it is interesting to see how completely in those days men's minds were under the control of authority—how little the right of private judgment had invaded even the precincts of science. I do not propose to trouble you with an exposition of Galenism in general; but it may not be without instruction, and I trust not without some little interest, for a few moments we examine what was the teaching of the great Pergamenian, in order that the reforma ion brought about by Harvey, the Luther of Medicine, may stand out in all the brilliancy that belongs to it, and with which it has illumined, and will ever continue to illumine, the path of genuine inductive science.

Galen bases his doctrine concerning the circulation on the dictum of Hippocrates, that the liver is the root of the veins, as the heart is of the arteries, and that from these organs the blood and the spirit pass to the different parts of the body, and warmth is diffused through them. From both he says that warmth flows through the body. The arteries which are in the heart correspond in size to the vei[illegible] in the liver; and in the same way as the artery which is given off from the left side of the heart constitutes the trunk of the arteries which occur throughout the living body, so the veins which are spread over the whole body spring from the vena cava, as branches from the trunk of a tree. The arteries which are distributed from the heart to the lung correspond in size to the veins which occupy the stomach, spleen and mesentery, and so on. This may serve as a specimen of Galen's anatomical notions regarding the vessels. I gather from other parts of his works what his views were regarding the functions of the vessels and the blood. The following passage is essentially all that he says upon what we should call the circulation, but concerning which, whatever faint indications may be traced of the doctrine in Galen or his successors now that every tyro has the facts properly propounded to him, no one possessed any real knowledge until demonstrated by him who, a hundred years after the condemnation of Dr. Geynes, was called by the College of Physicians "ubique amor et desiderium."

"The mesentery," we find Galen writing, "supports two veins which emerge beneath the liver, and they subsequently are distributed into various parts, and are inserted into the mesentery, by which means, as they are thin, they escape the danger of rupture. They adapt themselves to the bending, and lying like leech[illegible]s, gaping with an open mouth upon the j[illegible]junum, suck up all the aliment which has been converted into juice in it. As soon as they have absorbed, they change into blood what in the jejunum was a liquid. The two veins that have arisen out of many, carry off what they have received from the mesentery by the gates that have already been mentioned. In the liver the food puts on the nature of blood, at the same time the various excrementitious matters are thrown off. Yellow bile is carried to the liver which is attached to it, black bile to the spleen. The food converted into blood is conveyed from the liver to the right side of the heart. From the heart the blood passes by the jugular veins to the upper parts of the body. By the vena cava it is poured through the entire body. From the hollow vein two branches pass, after the blood has been separated from the serum to the kidneys, which lie under the loins towards the hips, and through which the urine transudes." The importance of the liver as the main organ of sanguification and as the source from which the blood springs, is often dwelt upon; all the blood takes its origin from the liver.

To us, with our present lights, all this appears only a monument of human ignorance; and yet, with such an anatomical and physiological nightmare weighing upon them, our learned ancestors who doctored the sick between the second and seventeenth centuries of the Christian Era, wrote prescriptions with probably as much, if not more, self-possession and self-confidence as our Todd's, Bright's, Latham's or Watson's of the present day. The difficulty of reconciling the discrepancies and incongruities that must have prevaded Galen's mind becomes greater when we find him explaining facts by hypotheses. Thus if you will bear with me and Galen a little longer, the arteries were to him, as you know, vessels that held a spirit or vapor; he assumed a power flowing from the heart to each artery, by which it dilates and contracts; and "because everything that dilates draws to itself from surrounding parts," he concludes "that those arteries which terminate in the skin draw air into themselves, and that those which at some part open to the veins draw the most refined and spirituous part of the blood which they contain to themselves." At the same time we meet with the admission that blood does get into the the arteries, but, it is supposed, to serve only for the nutrition of the arterial coats. Again we find him speaking of ateriotomy as a means of emptying both veins and arteries, "which cannot well happen, he says, "unless the anastomose with one another." Again, the effect of a ligature upon an artery was well known to Galen, for he observes, that if arteries be constituted by a ligature at any part, the part above the ligature nearest the heart is seen to beat as before, while the part that is below is at once deprived of the pulse; it is manifest that this movement proceeds from the heart to the arteries.

Is it not strange that, being so shrewd an observer, he should have allowed his mind to become warped by spurious theories, so as not to penetrate a little further into the mystery of the circulation? and is it not a still greater enigma, that with Galen's example as a dissector and vivisector and pathologist, the medical fraternities of fifteen centuries should have failed to look for themselves, and should have preferred groping in the murky fog of authority to enjoying the bright light of inductive science?

The period of incubation was indeed a long one. Before the birth there were not the usual phenomena and disturbances which so often indicate the coming event. Together with the "novum organon," the great "exercitatio de motu cordis," which has cast Galenism to the winds, and inaugurated a new phase of the world's growth, sprang from the brain of Harvey like a new Athene, in complete and gorgeous panoply, from the head of Zeus.

The little man, of lowest stature, round-faced, and of olivaster complexion, with the small, round, black eye, full of spirit, and with hair black as the raven's coat—such is the description given us of Harvey—did not arrive at his conclusion without much and hard labor; but of all the works in medical science which emb[illegible]dy great and important truths promulgated in them, for the first time, there is none to which in every sense the "teres atque rotundus" of Horace more completely applies than to the "exercitatio de motu cordis." There is a completeness of proof, a certainty of conviction, and an absence of dogmatism in that small volume which, as Dr. Mead truly says, laid the foundation for rational medicine; that it will ever serve as a model to the medical writer, as it will remain a memorial of one of the greatest intellects which has ever lived.

There are few things more grateful to the thoughtful mind than to find an opportunity to dwell upon the achievments of our forefathers, and to pay to them, at fitting times, the debt which we owe, but which in the turmoil of life we but too often overlook and forget. You will the more readily, I believe, acknowledge the deep obligation which we, as men of science, and our fellow men at large, are under to William Harvey, if you will compare, for a moment, the absurd mystification which Dr. Geynes was sworn to believe in, and the broad facts of the circulation which are now known to the first year's student, and which form the very groundwork of our daily practice. Should there be any who have not yet read the exercitation, let me beseech you in justice to yourselves, no less than in justice to him from whom this society takes its name, not to let the week go by without having afforded yourselves the treat that has been too long denied you. Dr. Willis's excellent translation for the Sydenham Society brings it home to us all, and you will not regret the hour you may devote to it.—*Annual Address Delivered to the Harveyian Society, by Edward H. Sieveking, M. D.*

